# Evaluation of the effects of different groove length and thickness of the retainers on the retention of maxillary anterior base metal resin bonded retainers-an in vitro study

**DOI:** 10.4317/jced.50714

**Published:** 2012-04-01

**Authors:** Anoop Nair, K M. Regish, N P. Patil, D R. Prithviraj

**Affiliations:** 1 MDS, Senior lecturer, Dept. of Prosthodontics. Govt. Dental College and Research Institute, Bangalore. Victoria Hospital Campus, Fort, Bangalore, India. 560002; 2 BDS (MDS),Post Graduate, Dept. of Prosthodontics. Govt. Dental College and Research Institute, Bangalore. Victoria Hospital Campus, Fort, Bangalore, India. 560002; 3 MDS, Principal, Jaipur Dental College, Rajasthan, India; 4 Professor and Head, Govt. Dental College and Research Institute, Bangalore. Victoria Hospital Campus, Fort, Bangalore, India. 560002

## Abstract

Objectives: The resin-bonded fixed partial dentures have gained immense popularity in recent years as they are more conservative, esthetic, economic and easily fabricated. However debonding is considered the most common cause of failure of resin bonded prosthesis. The objective of the study were to compare the effects of different groove lengths and thickness of retainers on retention of maxillary anterior base metal resin bonded retainers. Study Design: Twenty five metal dies of maxillary central incisor duplicated from pure typhodont teeth (maxillary left central incisors) prepared to receive retainer for resin bonded fixed partial denture having different test designs were made. Five test groups were made with each group having five specimens. Two groups were having preparation depth of 0.5mm and retention groove lengths of 3mm and 5mm. Two groups had preparation depths of 0.3 and 0.7mm with no groove preparation with retainer thickness of 0.3 and 0.7mm respectively. Fifth group with no groove preparation and preparation depth of 0.5mm was kept as control for all the groups. All the specimens were cemented using calibra (Dentsply) resin cement. Each specimen was subjected to tensile loading in vertical direction on universal testing machine (Instron 5569) at a crosshead speed of 1mm/min. \ Results: Groups with 5mm and 3mm groove length recorded higher mean vertical forces when compared to the group with no groove preparation. Group with 5mm groove length showed highest mean vertical forces. The group with 0.7mm retainer recorded higher mean vertical force values when compared to groups with 0.5mm and 0.3mm retainer thickness. Group with 0.3mm retainer thickness recorded the least mean vertical force value. Conclusion: Placement of the grooves increased the retention values almost 2 ½ times than the grooveless preparation and as the thickness of the retainers increased retention values also increased. Retention value was directly proportional to the groove length and retainer thickness.

** Key words:**Groove length, retainer thickness, resin bonded bridges.

## Introduction

Conventional procedures for the preparation of abutment teeth often involve major removal of tooth structure. If coverage is necessary for cosmetic purposes because of caries or preexisting restorations, this removal of structure is acceptable. However when the abutment teeth are sound, conventional full coverage procedures seem quite radical. More conservative procedures, such as partial veneer crowns or pin-retained castings, present limitations in esthetics and retention(1), that led to the development of a technique for the fabrication of fixed partial dentures, involving little or no preparation of abutment teeth.

Resin bonded cast metal fixed partial denture have become an alternative to the conventional fixed partial dentures in the last two decades. Dental luting cements serve as a link between indirectly fabricated restorations and the prepared tooth surface (2). In addition to sealing of the micro gap present between the metal and the abutment tooth, the adhesive cements greatly contribute to the retention of the prosthesis, as the amount of tooth coverage is less.

Thickness of the retainer used for such prosthesis, which is under operator´s control, can be easily increased and hence increase the overall strength and retention of the prosthesis. However more the thickness, it will require the tooth structure to be reduced more, therefore the optimal thickness needs to be determined. Previous studies have used empirical thickness of metal between 0.3and0.5mm (3,4), these thicknesses of base metal alloy retainer were considered adequate to resist distortion during function, but data available is not enough to support or contradict this assumption.

Sparse research has been conducted to investigate the influence of different retainer design (groove length) or depth of abutment preparation on retention of resin-bonded prosthesis. In view of the different designs as to which is the best method of providing retention to the resin bonded prosthesis, the present study evaluates the effect of different proximal groove lengths and thickness of retainers on the retention of maxillary anterior base metal resin bonded retainers.

This study is an effort to assess the contribution of length of proximal grooves and thickness of base metal retainers on the retention of resin bonded retainers.

## Material and Methods

Preparation of typhodont teeth and metal dies

**Figure 1:**
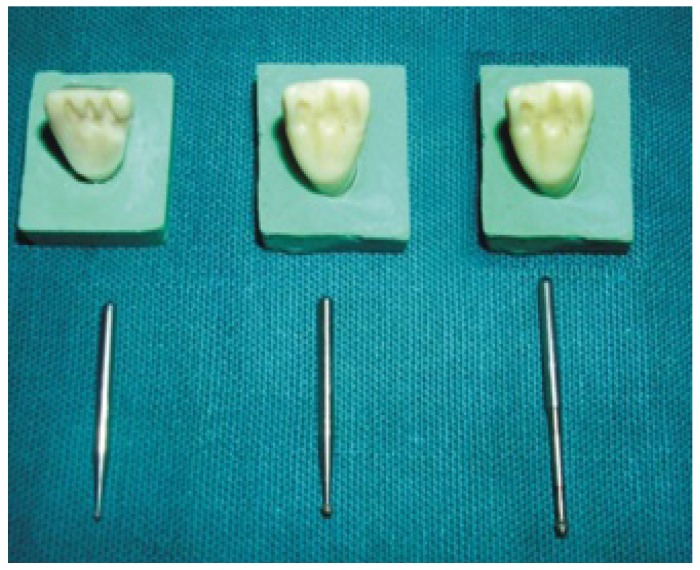
The depth of preparation was kept with the help of depth orientation grooves made with 1mm and 0.7 mm bur.

**Figure 2:**
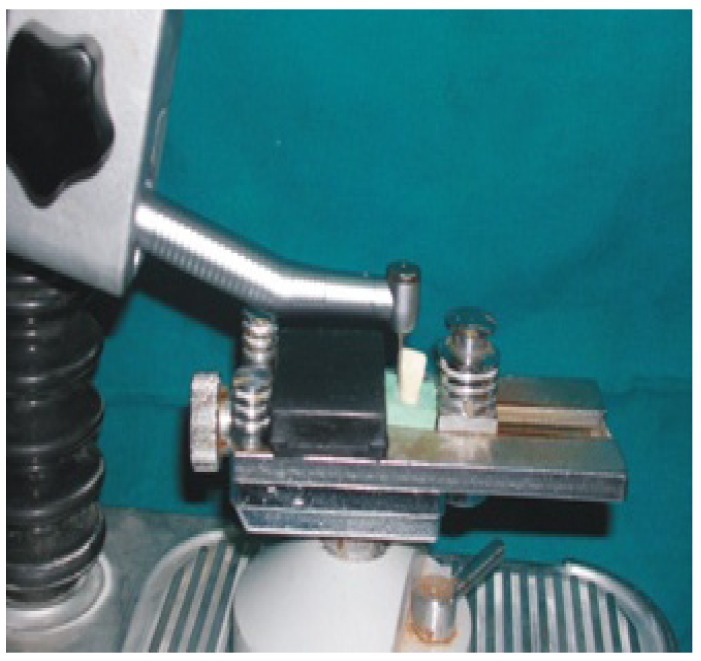
K-9 (KAVO).

Five maxillary central incisor Typhodont teeth (kulzer Germany) were used for the purpose of this study. Palatal surface of three central incisors were reduced 0.5mm in depth with the help of round bur. Preparation was extended proximally terminating just lingual to the facioproximal line angle to increase the tooth surface area for bonding and to allow a definite path of insertion, almost similar to that of partial veneer crown. The depth of preparation was kept 0.5mm with the help of depth orientation grooves made with 1mm bur. The fourth incisor tooth is prepared as above except depth of preparation was kept at 0.3mm where as fifth incisor had 0.7mmdepth of preparation (Fig. 1). Two incisors with 0.5mm depth of preparation were used for placement of proximal grooves. The grooves were prepared so that they were parallel to each other and to the path of insertion this was achieved by using K-9 (KAVO) crown finishing installation to which the hand piece was attached (Fig. 2). The lengths of the axial grooves were kept 5mm for one tooth specimen and 3mm for other specimen.

All the five specimens were then duplicated using hard inlay wax and converted into metal dies.

Fabrication of metal retainers

**Figure 3:**
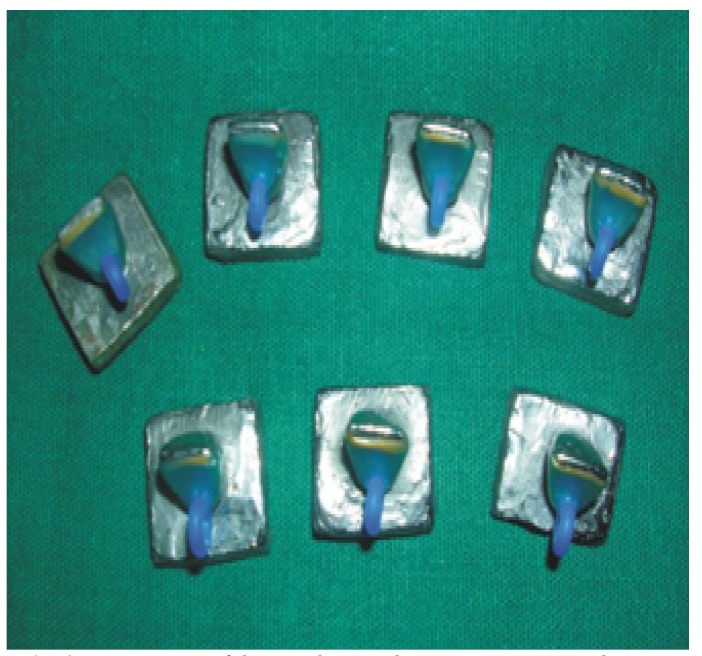
Wax patterns fabricated using direct wax pattern technique directly over the metal dies.

Pattern wax (Renfert, Schuller Germany) of 0.3mm, 0.5mm, 0.7mm thickness was used for fabrication of wax pattern. Wax patterns were fabricated using direct wax pattern technique directly over the metal dies to avoid distortion during the removal of pattern from the die, which could induce errors in the thickness of the patterns (Fig. 3). An inverted U shaped wax loop was made up of 2mm round sprue wax which was attached on the palatal surface over the cingulum area, which would later serve as an attachment for loading on the universal testing machine.

Cast on technique was planned for the casting of the retainers, as removal of the patterns would lead to distortion and variations in setting expansion, thermal expansion, marginal inadequacies and fit of castings could be eliminated. Likewise, in total twenty-five metal retainers casting were obtained following the same procedure.

Calibra resin cement (Dentsply) was used to bond the retainers to the metal dies.

All twenty-five samples were tested for vertical dislodgement using Instron universal testing machine (Instron UTM, model 5569).

The values obtained were noted and recorded in Kilo-Newton (KN), which formed the basic data of the study.

Mean and standard deviation of the dislodging forces were determined and subjected to statistical analysis. ANOVA test was employed to determine if there were differences in the mean dislodging forces among the various groups.

Student t- test was also employed for pair wise comparison in the mean dislodging forces among the various groups.

## Results

The values for the tensile force required to dislodge the base metal retainers were recorded in kilo Newton´s (KN) which forms the basic data of the study (annexure). The values obtained were tabulated and converted using the following formula 1KN=1000N

The results reveal that the highest mean vertical retentive force was recorded for the group with groove length of 5mm (251.88 N ± 27.86) and the least for group with no groove preparation (90.97 N ± 17.64). Group with groove length of 3mm showed vertical retentive force value (211.72 ± 19.55) almost similar but less than group with 5mm groove length. Almost 2 to 2 1/2 times more resistance to dislodgement was observed in retainers with 3mm and 5mm groove length on central incisor preparation as compared to group of specimens with no groove preparation. The results showed that thicker retainers required more force to dislodge from the metal die.

The results reveal that the highest mean vertical retentive force was for group with retainer thickness 0.7mm (117.6 ± 21.57) and the least was for the group with retainer thickness 0.3mm (45.88± 9.93). Group with 0.5mm retainer thickness which was kept as control showed almost similar but less vertical retentive force (90.97 ± 17.64) as compared to group with 0.7mm retainer thickness. The percentage increase in dislodging forces were greatest between 0.3 and 0.5mm thickness around 50% when compared to 0.5 and 0.7mm thickness which showed minimal increase in dislodging forces.

## Discussion

Anterior resin bonded fixed partial dentures are largely dependent on the actual surface area of the enamel covered by the retainers for retention. (3) A maximum area of etched enamel may be difficult to achieve in the anterior region without a display of the interproximal metal. Ferrari et al (5) reported 0.2mm to 0.4mm enamel thickness at the gingival third of the lingual surface of anterior teeth. Exposure of dentine in some regions may be unavoidable during tooth preparation, particularly when the abutments have wear facets. Limited interocclusal space and a thin translucent incisal edge are also factors that may limit the surface area for bonding.(6) Proximal grooves have been recommended by certain investigators to compensate for the lack of proximal wrap around to improve the long term prognosis of anterior resin bonded fixed partial dentures.(3,7,8)

This study was planned to evaluate the tensile (retentive) force required to dislodge resin bonded retainers from metal dies with different groove lengths and the force was directed along the path of insertion. The results of the study indicated that addition of proximal grooves to the original abutment design of anterior resin bonded bridges resulted in substantial increase in retention.

 Table 1 show means and standard deviation of vertical retentive forces of groups with different groove lengths of 3mm and 5mm whereas 0mm acted as a control (i.e. no groove preparation). The results revealed that the highest mean vertical retentive force was recorded for the group with groove length of 5mm (251.88 N ± 27.86) and the least for group with no groove preparation (90.97 N ± 17.64). Group with groove length of 3mm showed vertical retentive force value (211.72 ± 19.55) almost similar but less than group with 5mm groove length. Almost 2 to 2 1/2 times more resistance to dislodgement was observed in retainers with 3mm and 5mm groove length on central incisor preparation as compared to group of specimens with no groove preparation. These findings were in agreement with those reported by Saad, Claffey et al (9) However, the mean loads required to dislodge the specimens of various groups in this study were lesser than those of Saad et al who also tested, the affect of groove placement on the retention of cast metal resin bonded retainers on metal dies. These variations may be contributed to the difference in methodology because of use of different resin cement (Calibra resin cement was used in this study instead of Panavia EX), difference in the degree and intensity of polymerization, film thickness of resin cements.

**Table 1:**
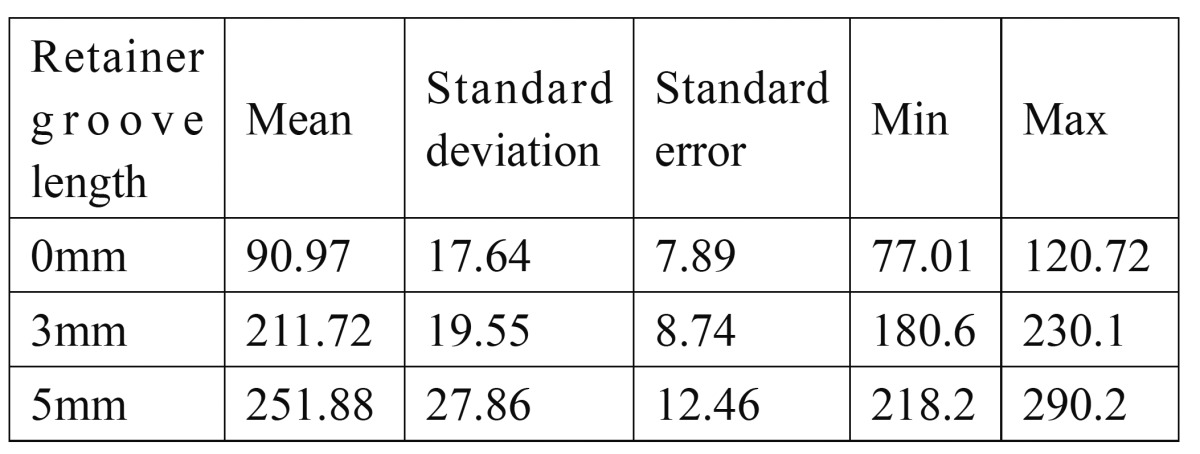
Mean and Standard deviation of vertical retentive forces(Newton) required to dislodge the resin-bonded retainers having different groove lengths.

Burgess and McCartney (7) reported similar results who tested the bond strengths of various designs of cast metal resin bonded retainers of intact extracted anterior teeth. The values were lower than the mean figures reported in this study, these differences may be explained by morphologic differences of the teeth in the two studies or by fractures of natural teeth. A lower mean value was reported for conventional partial veneer crowns with incisal and proximal grooves by Lorey and Myers (9), who used metal dies and zinc phosphate cement. Murkami and Barrack (10)tested bond strengths of etched cast resin-bonded retainers cemented with resinous cement (Conclude 3M) to natural teeth. They reported an increase in mean peak failure load for maxillary anterior incisors designed with a V shaped cingulum rest and proximal extension. Williams et al (11) reported a mean dislodging force for perforated metal retainers provided with 16 (0.5 mm diameter) holes and cemented with composite resin cement to extracted human teeth. The results in these studies were consistent with those reported in this study.

 Table 2 shows the statistical pair wise comparison between groups having different groove lengths by studentt test of mean vertical (retentive) dislodging forces. These results showed statistically significant difference between control (0mm) groove and 3mm groove (t= -10.25, p=0.0000 at 1% level of significance), control (0mm) and 5mm groove length (t= -10.90, p=0.0000 at 1% level of significance), 3mm and 5mm groove length (t= -2.63, p=0.0298). This improvement in retention of cast metal resin bonded retainer reported in this study for grooved specimens with increasing length could have been achieved by increasing surface area and by mechanical locking. Murkami and Barrack (10) reported a linear relationship between the surface area for bonding and the force required to debond resin bonded fixed partial dentures. However Potts et al (12) and Kishimoto (13) et al reported that addition of proximal grooves did not substantially improve retention of conventional partial veneer restorations.

**Table 2:**
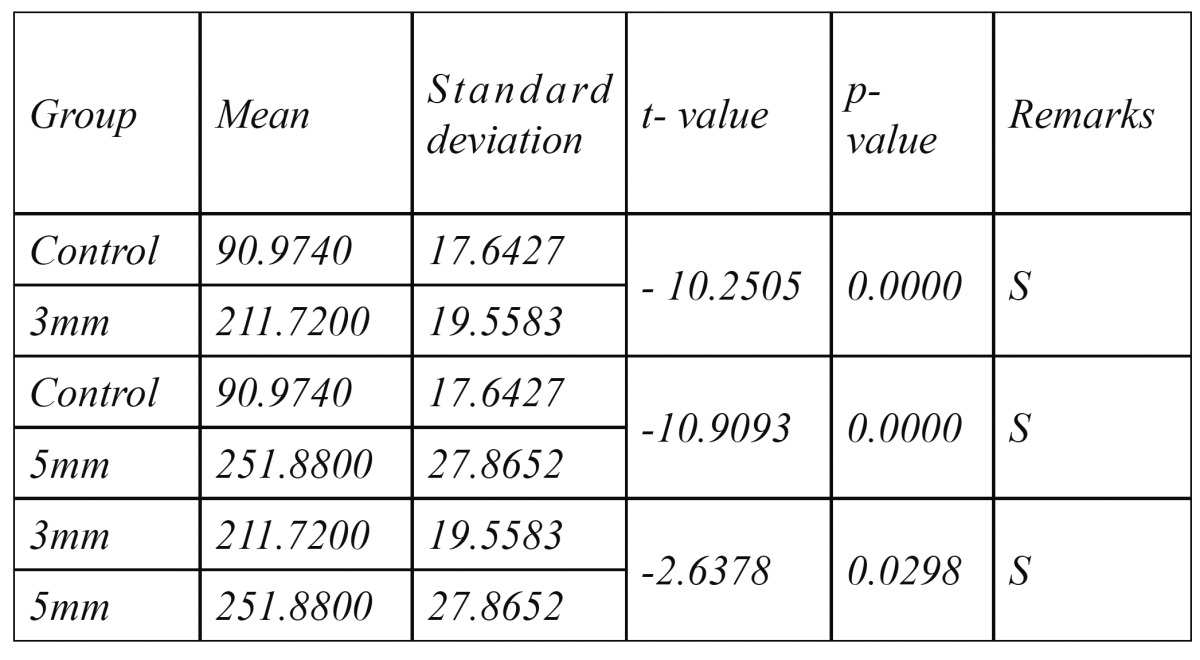
Statistical pair wise comparison (Student - t test) of Dislodging Forces (N) of groups with different groove lengths and no groove. i.e. control group (n=5).

The results in this study showed that thicker retainers required more force to dislodge from the metal die.

 Table 2 and  Table 3 and show means and standard deviation of vertical retentive forces of groups with different retainers thickness (0.3mm, 0.5mm and 0.7mm). The results revealed that the highest mean vertical retentive force was for group with retainer thickness 0.7mm (117.6 ± 21.57) and the least was for the group with retainer thickness 0.3mm (45.88± 9.93). Group with 0.5mm retainer thickness which was kept as control showed almost similar but less vertical retentive force (90.97 ± 17.64) as compared to group with 0.7mm retainer thickness.

**Table 3:**
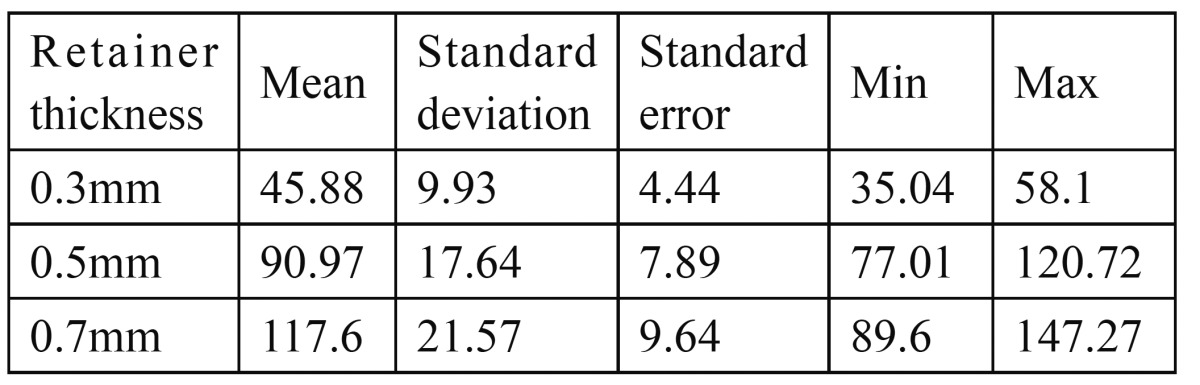
Mean and Standard Deviation of vertical retentive forces (Newton) required to dislodge the Resin-bonded retainers having different retainer thickness.

The percentage increase in dislodging forces were greatest between 0.3 and 0.5mm thickness around 50% when compared to 0.5 and 0.7mm thickness which showed minimal increase in dislodging forces. These findings were in agreement with Ibrahim et al (4) who in their study reported an increase in retentive forces as the retainer thickness was increased.

 Table 4 shows the pair wise statistical comparison between groups having different retainer thickness by students ´t´ test of mean vertical (retentive) dislodging forces. These results showed statistically significant difference between (0.5mm) and 0.3mm retainer thickness (t=4.97, P=0.0011) between 0.5mm and 0.7mm retainer thickness (t= -2.13, P=0.0651), between 0.3mm and 0.7mm retainer thickness (t = -6.75, P=0.0001).

**Table 4:**
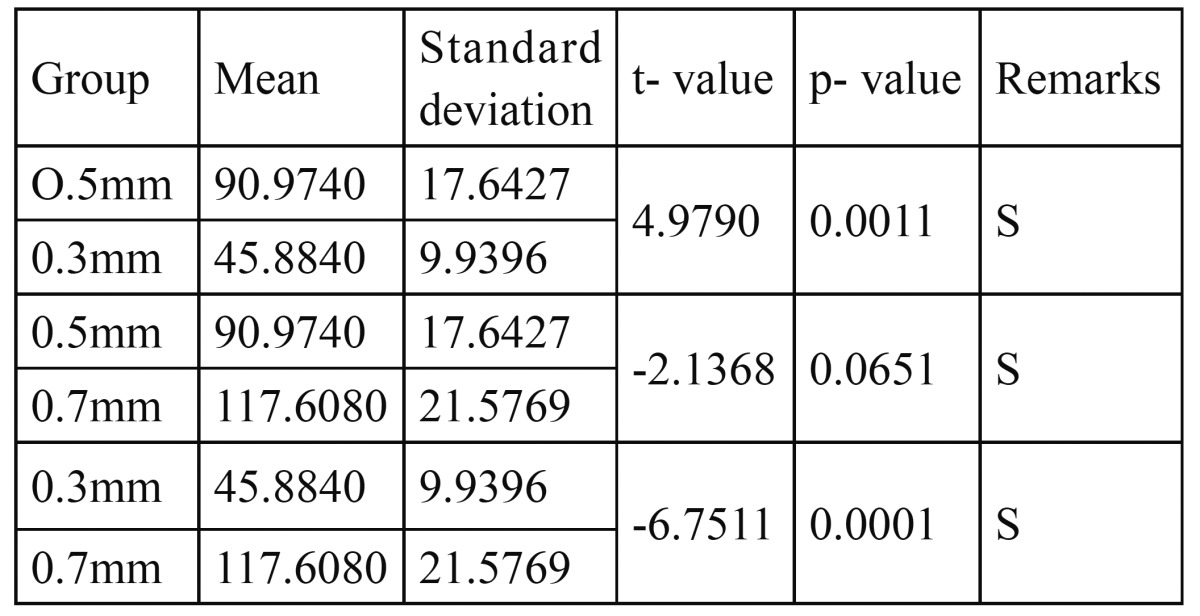
Statistical pair wise comparison (Student - t test) of the Dislodging Forces (N) of groups with different retainer thickness. (n=5).

Sato et al (14) evaluated the effects of thickness and Young´s modulus of elasticity on resin bonded retainers, with a three dimensional finite element analysis with selected loading conditions. These researchers demonstrated that the failure probability diminished as the thickness of cast metal retainers increased and that metals with a low Young´s modulus were more sensitive to metal thickness.

The thickness of the retainer is directly influenced by the occlusion, enamel removal before exposing dentin, and development of over contouring with subsequent periodontal problems. Ferrari et al (5) listed an anatomic guide for reduction of enamel for resin bonded prosthesis and suggested reducing the enamel of 0.2 to 0.4mm. However from the data of his study, enamel reduction may not be sufficient to allow adequate thickness of the cast metal to provide optimal retention.

However this study suggested that retainers made from alloys with a high modulus of elasticity can also benefit from retainer thickness ? 0.5mm and any thickening of retainer should be balanced against the problems brought about by clinically removing more tooth structure and an over contouring of the lingual surface.

## Conclusion

Within the limits of the present study and based on the findings of this study and its statistical analysis it has been concluded that

1. Placement of the grooves increased the retention values which are almost 2 1/2 times more for the preparation having 5mm groove length as compared to the test specimen having no groove preparation. Upon statistical analysis(using ANOVA and student t test ) statistically significant values were observed for all the groups, in between the groups when compared with control group having no preparation, i.e. retention values was directly proportional to the groove length.

2. The retention values increased almost two times i.e. from 0.3mm retainer thickness to 0.5mm retainer thickness. considerable increase in the retention values was observed when the thickness was increased from 0.5mm to 0.7mm.The increase in the thickness of the retainer resulted in higher retention values for thicker specimens(0.7mm thickness) as compared to the control group(0.5mm thickness)were as the decrease in thickness(0.3mm thickness)has shown less retention values as compared to the control group(0.5mm thickness).

3. Though the retention values increased with the increase in groove length as well as increase in the thickness of the retainers there was positive co relation between groups having 3mm and 5mm groove lengths with the group having 0.3mm retainer thickness, except for the group having 0.7mm retainer thickness which showed negative co relation.
